# Occupational therapy‐led environmental assessment and modification—A quantitative evaluation of a pilot implementation study in a regional/rural setting

**DOI:** 10.1111/1440-1630.70039

**Published:** 2025-07-14

**Authors:** Alison Pighills, Susan Melchert, Tessa Brondello, Alicia Eden, Erin Rickman, Anna Tynan

**Affiliations:** ^1^ School of Occupational Therapy, College of Healthcare Sciences James Cook University Douglas Queensland Australia; ^2^ Mackay Institute of Research and Innovation Mackay Hospital and Health Service Mackay Queensland Australia; ^3^ Community Health and Therapy Services Mackay Hospital and Health Service Mackay Queensland Australia; ^4^ Research Development and Engagement Unit, Baillie Henderson Hospital Darling Downs Health Toowoomba Queensland Australia; ^5^ Southern Queensland Rural Health The University of Queensland Toowoomba Queensland Australia; ^6^ The Centre for Health Research University of Southern Queensland, Springfield Campus Springfield Central Queensland Australia

**Keywords:** environmental assessment and modification, fall prevention, home assessment, implementation, occupational therapy

## Abstract

**Introduction:**

Environmental assessment and modification is an effective fall prevention intervention for high‐risk older people, which has not yet been adopted in routine occupational therapy practice.

**Methods:**

A pilot pre‐post quantitative implementation study was carried out in a regional hospital and health service, within a community‐based occupational therapy service, in Queensland between 2019 and 2022, involving 279 occupational therapy medical chart audits of people aged ≥65 at high risk of falls, clinical practice observations of 9 participating occupational therapists, and 32 staff questionnaires.

**Consumer and Community Involvement:**

Occupational therapists were included as co‐researchers, but there was no health consumer or community involvement in the study.

**Results:**

Baseline fall rates were higher than anticipated, with 71% of clients sustaining ≥1 fall in the year preceding intervention and 52% sustaining ≥2 falls. Sixty per cent of people with ≥3 of the 5 identified fall risk factors died during the study period. Implementation outcomes included penetration, fidelity, acceptability, and sustainability.

**Penetration**

A fall prevention environmental assessment was provided when indicated in 80% of cases during and immediately after implementation. This increased to 97% 2 years after implementation.

**Fidelity**

When intervention was indicated, the occupational therapists delivered a high‐intensity fall prevention environmental intervention, as per protocol, in 100% of cases.

**Acceptability**

Staff surveys revealed a marginal, non‐statistically significant, increase in staff knowledge, attitude, confidence, and experience in fall prevention between the baseline and follow‐up periods.

**Sustainability**

Medical chart audits revealed that implementation was sustained for 30 months and beyond post implementation.

**Conclusion:**

This project demonstrated that environmental assessment and modification was delivered per protocol, embedded in clinical practice, and sustained after implementation. Occupational therapists' precision, in determining who should receive the intervention, increased over time. The results of this pilot will inform the design of a national implementation study, which commenced in 2025.

**PLAIN LANGUAGE SUMMARY:**

Checking an older person's home for fall risks can help lower their chances of falling by about 38%, especially if they are more likely to fall. People may be at higher risk because of how they do certain tasks, the tasks themselves, hazards in the environment, health issues, or a mix of these things. An occupational therapist does the assessment by watching the older person do daily tasks, like getting dressed or doing chores. This helps to find out what might cause a fall. The therapist then talks with the person about which activities they feel are most risky, asks for their ideas on how to stay safe, and helps them to make a plan. The therapist also checks in later to help put the plan into action. In this study, we trained therapists to do this kind of fall check. We looked at whether occupational therapists followed the training, if the right people were offered the assessment, and if it was still used after the project ended. We found that therapists did the assessments as they were trained to and with the people who needed them most. The therapists also kept doing the assessments after the project was over. This likely helped reduce falls.

**Key Points for Occupational Therapy**

- Occupational therapist‐led environmental assessment for fall prevention is a clinically effective intervention.
- Good practice guidelines recommend using an assessment tool validated for fall prevention and providing follow‐up.
- Fall‐specific environmental assessment and modification is not routinely provided, but it can be successfully implemented and sustained.

## 1 INTRODUCTION

Falls are pervasive among older people, affecting around 35% of those aged 65–79 and 50% of people over 80 (Campbell & Robertson, 2007; Gillespie et al., 2012). They are attributed to 43% of both injury‐related hospitalisations and deaths in Australia, accounting for $4.7 billion in annual hospital costs (Australian Institute of Health and Welfare, 2025). Falls cause serious injury and are a common antecedent to institutionalisation via a vicious circle of fear of falling, activity avoidance, and functional decline (Pighills et al., 2024).

Environmental hazards are a major contributor and are estimated to cause 30%–50% of falls among older people (Rubenstein, 2006). Prevention strategies, such as home environmental assessment and modification (EAM), can reduce falls by around 38% when targeted towards high‐risk populations and provided by occupational therapists (Clemson et al., 2008, 2023; Gillespie et al., 2012; Pighills et al., 2011).

EAM for fall prevention involves a systematic functional environmental assessment aimed at home fall‐hazard reduction, awareness raising, and joint problem solving with the older person. It takes the form of comprehensive assessment, using a valid, reliable assessment tool, such as the Westmead Home Safety Assessment (WeHSA), which takes 1.5 hours to complete (Clemson, 1997). The assessment considers the older person's intrinsic risk, their environment, and the activities of daily living (ADLs) in which they engage, with interventions consisting of person‐, environment‐, and activity‐focussed (or occupation‐focussed) strategies (Gitlin, 2009; Pighills et al., 2016; Stevens et al., 2014).

Due to its effectiveness, EAM has been incorporated into national and international fall prevention guidelines (Australian Commission on Safety and Quality in Health Care, 2025; Clemson et al., 2023; Montero‐Odasso & van der Velde, 2022). However, this intervention has not been routinely adopted in occupational therapy clinical practice (Clemson et al., 2014; Pighills, Furness, et al., 2019; Tynan et al., 2022).

Some research has focussed on perceptions of evidence‐based practice (EBP) in rural settings, in both Australia (Bennett et al., 2003) and the United Kingdom (Hu, 2012); however, minimal research exists investigating implementation strategies to enhance uptake of best practice by occupational therapists in regional and rural locations. Furthermore, despite implementation being recognised as a research priority for occupational therapy (Juckett et al., 2019), we found minimal existing implementation research on the uptake of environmental interventions to prevent falls, particularly in the rural and remote context.

**FIGURE 1:**
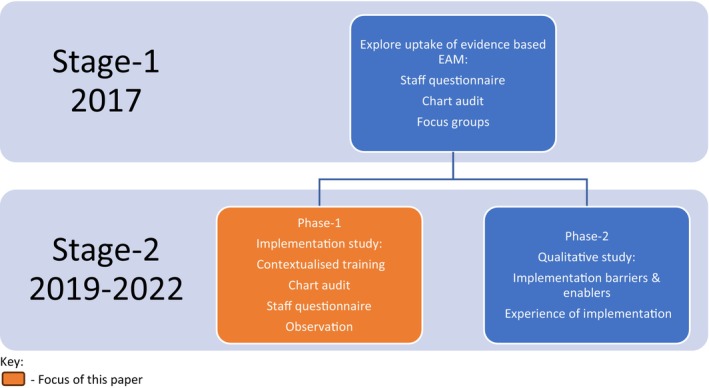
Project stages—Translating Research into Practice (TRIP)—occupational therapist‐led environmental assessment and modification (Pighills, Furness, et al., 2019; Tynan et al., 2022).

We have undertaken a programme of research exploring the uptake of EBP, implementation barriers and enablers, and an implementation evaluation over two stages (Figure 1). Stage 1 examined clinicians' knowledge, confidence, attitudes, and experience of providing EAM for fall prevention (questionnaires); current practice (chart audits); and determinants of implementation (focus groups) (Pighills, Furness, et al., 2019). Despite self‐reported knowledge, confidence, and experience in EAM, the audited charts revealed that this intervention was not delivered in practice. Influences on uptake of EAM included confidence in and awareness of research evidence, stakeholder support, and perceived impact of limited resources in the rural context. Although occupational therapists were aware of, and experienced in, fall prevention intervention in the home, there was no evidence of them implementing best practice EAM (Pighills, Furness, et al., 2019).

In Stage 2, we carried out an implementation project. The project aimed to support occupational therapists to change practice (in line with clinical guidelines), standardise service delivery, develop an implementation plan and contextualised training materials (based on identified barriers and enablers), and evaluate the implementation outcome by answering the following research question: Can community health occupational therapists, in a regional/rural area, adopt, implement, and sustain best practice EAM to reduce fall risk in community‐dwelling older people and act as mentors to other occupational therapists within the area?

**FIGURE 2:**
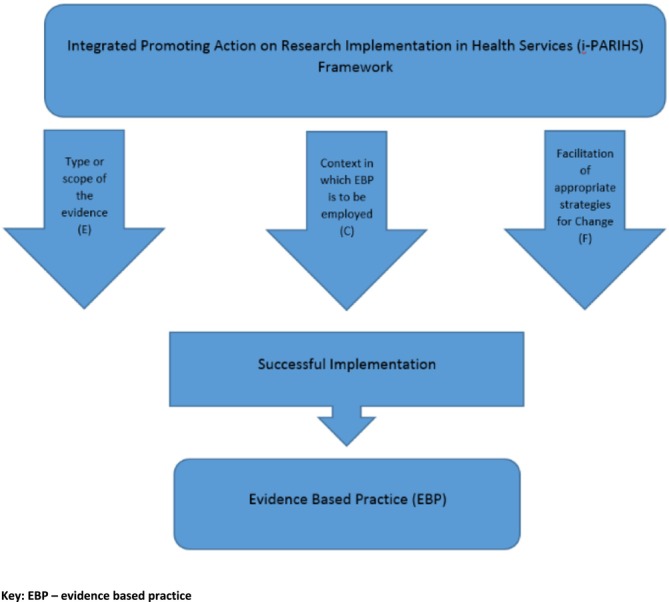
Implementation of evidence into practice—the i‐PARIHS framework.

This translational research project (stage 2) used the Integrated Promoting Action on Research Implementation in Health Services (i‐PARIHS) framework to guide the translation of evidence into practice (Figure 2). The i‐PARIHS conceptual framework is widely used for embedding EBP. The i‐PARIHS framework posits that successful implementation (SI) is dependent on four main components—facilitation (Fac^n^), innovation (I), recipients (R), and context (C). Facilitation is the active ingredient and involves understanding and adapting characteristics of the innovation (I) for the recipients (R) of the innovation. Specifically, the i‐PARIHS framework identifies critical elements for implementation (I, R, and C) and guides the implementation strategy (Fac^n^) to achieve successful and sustained change (Harvey & Kitson, 2016). This framework engages stakeholders in identifying critical aspects of implementation and the nature of needed change. Significantly, this framework argues that SI has as much to do with the recipients, the context, and the facilitation as it does with the quality of the evidence.

The stage 2, phase 2 qualitative investigation of the barriers, enablers, and experience of implementation of best practice EAM for regional and rural occupational therapists was carried out across two public health services in Queensland, within community‐based occupational therapy services (reported elsewhere) (Tynan et al., 2022). It found that opportunity (factors outside of the individual's control) was the main limitation influencing implementation due to team structures, multiple responsibilities, differences in access to resources, and lack of connection between services. It also revealed that, despite similar beginnings, one site ceased implementation, whereas the other reported continued success and implemented and embedded the evidence‐based intervention.

This paper aims to complement the qualitative review by focusing on the quantitative evaluation of the implementation of EAM at the successful site and examining the sustainability of the practice change. Understanding the implementation process, outcome, and sustainability is key to identifying effective approaches to implementing evidence‐based interventions. Examining the influence of context as a critical driver can allow clinicians to better understand and overcome barriers to implementation.

This stage 2, phase 1 quantitative analysis and the phase 2 qualitative study (Tynan et al., 2022) will form the basis for further enquiry into the interplay between the practice change, the context, and the recipients, as significant components of SI and sustainability of innovation in health care (Harvey & Kitson, 2016).

## 2 METHODS

### 2.1 Positionality statement

All The authors of this journal article identify as Caucasian female occupational therapists, with varying levels of engagement in fall prevention. All share a deep understanding of, and empathy for, the personal cost of falls. Authors 1 and 6 have clinical and fall prevention research backgrounds, spanning decades. Author 2 is a Doctor of Philosophy candidate with a clinical and higher education background, and Authors 3–5 are clinicians. As occupational therapy clinicians, educators, and researchers, we seek to address the research–practice gap in this field, which has shaped the scope of this study.

### 2.2 Study design

A quantitative pre‐evaluation–post‐evaluation was conducted as part of the broader stage 2 implementation project.

During the planning phase, learnings from stage 1 (Figure 1) informed an implementation strategy, which was created following stakeholder discussions with organisational leaders (executives, directors of occupational therapy, and opinion leaders). The implementation strategy was refined using the Expert Recommendations for Implementing Change (ERIC) implementation strategies (Powell et al., 2015). The implementation strategy was iteratively refined, based on emerging findings from the concurrent qualitative study (Figure 1—stage 2, phase 2), which identified contextual influences on implementation (Tynan et al., 2022). An evaluation plan was produced using the logic model (Julian, 1997).

A local reference group of occupational therapists was identified to raise awareness, deliver the intervention, and provide future peer support to sustain the practice change. Site‐specific occupational therapy advisors agreed on the education and training plan, communication messages, and membership of the reference group and overarching steering committee. The overarching steering committee monitored implementation and was comprised of nominated leaders, managers, emerging opinion leaders, content experts, and researchers. The bi‐monthly reference group, comprising frontline staff and researchers, addressed local issues and identified implementation options.

### 2.3 Study setting

A public health service, covering an area of 90,000 km^2^ and providing acute and community‐based health care to 180,000 people living in regional and rural areas of Queensland, Australia, successfully implemented the practice change. Context was critical to implementation success (Tynan et al., 2022). The health service was geographically distant from metropolitan areas and thus may have suffered less disruption from the COVID‐19 pandemic, which delayed study implementation to 1 June 2020, with data collection from 1 June 2020 to 31 December 2022. One of the investigators, with experience implementing this intervention in other settings, was based at this site, which may have had a positive influence on take‐up, and the occupational therapy service had the advantage of previously working on an unrelated project with this investigator, so a working research relationship already existed. The site also had a defined community health team, which was the sole focus of implementation.

### 2.4 Intervention

Community occupational therapists, identified as the core group who would be most likely to assess home fall risk, were trained in the implementation of best practice EAM, with a view to them acting as mentors for other occupational therapists. The transition of the community occupational therapy service from their usual practice methods to the best practice intervention was completed within an action research cycle process of auditing, feedback from key stakeholders via focus groups and in‐depth semi‐structured interviews, consultation with the reference group to refine the implementation strategy, and introducing new implementation strategies.

Training on how to conduct EAM, including administration of the WeHSA, was available in a variety of modalities:

1. a 1‐day face‐to‐face training session delivered by the primary investigator;
2. an online training package, based on the WeHSA manual (Clemson, 1997): https://fallspreventiononlineworkshops.com.au/; and
3. observation of the intervention being delivered by a trainer with subsequent work shadowing of the trainee delivering the intervention and feedback from the trainer.

Following training and feedback from the reference group on operational changes needed, such as referral pathways, community health clients were identified as potentially at risk of falls by a participating occupational therapist, based on a review of the referral information. These clients were offered EAM to identify personal, environmental, and occupational fall‐related hazards and receive subsequent intervention. Based on existing evidence and the authors' previous experience in fall prevention research, stringent fall risk criteria were applied to the study, as the intervention is only effective in the high fall risk category (Clemson et al., 2023; Pighills et al., 2011). Fall risk screening was carried out on first contact. To be deemed high risk, clients had to be ≥65 years old, have sustained ≥2 falls in the preceding 12 months, and possess two out of the following three additional fall risk factors, which have been shown to produce the highest predictive risk scores for falls (Lusardi et al., 2017): (1) functional decline/assistance required with personal ADLs, (2) balance impairment/use of a mobility aid, and (3) fear of falling. Functional decline and balance impairment were identified through interview and functional assessment, and fear of falling was established by asking, ‘Are you afraid of falling?’. This simple question elicited a yes/no response and has good predictive validity (Denkinger et al., 2008).

The environmental assessment began with an initial discussion about the client's history of falling, lifestyle, patterns of usage of areas in the home, risk‐taking behaviour, strategies already adopted to reduce falls, environmental changes previously made, and an assessment of functional mobility, balance, and vision. This was followed by the environmental assessment using the WeHSA tool, a 57‐item functional assessment organised into 15 domains: external traffic ways, general indoors, internal traffic ways, mobility aid, pets, living area, seating, medication management, safety call system, bedroom, footwear, bathroom, toilet area, kitchen, and laundry. After assessment, the occupational therapist and client discussed identified potential fall hazards and possible solutions, and a list of recommendations was agreed. If possible, and in consultation with the client, identified hazards were addressed and rectified during the assessment. Carers were involved in the initial and post‐assessment discussions and functional assessment, if available. The occupational therapist made referrals to other agencies for equipment and modifications and liaised with the client and/or family members regarding recommendations for the private purchase of equipment (such as long‐handled aids and height‐adjustable rotary washing lines). The occupational therapist made a clinical judgement on whether a follow‐up visit was required. A written summary of recommendations was sent to, or left with, the client, and the occupational therapist or occupational therapy assistant made a follow‐up telephone call, as clinically indicated, 8–12 weeks after assessment to check the status of recommendations, monitor adherence, and provide further advice if necessary.

### 2.5 Evaluation and data collection tools

The pre‐post‐quantitative implementation evaluation comprised the following:

1. A baseline structured observation of current occupational therapy practice within the participating community health team to establish the level of adherence to existing evidence‐based guidelines and to understand contextual issues: During implementation, observations were carried out by peers to test fidelity to the intervention protocol, to identify whether changes needed to be made to how the intervention was delivered, to sustain the practice change through the supervision process, and to establish a ‘citizen's science’ approach to peer practice review and providing developmental feedback. An observation checklist was used, based on the domains contained in the WeHSA.
2. A chart audit, using a tool that included the key elements of best practice in EAM, was completed at baseline, during and after implementation. EAM was deemed high intensity if it included three of the following four components (Clemson et al., 2008):
  • a comprehensive process of hazard identification and priority setting, considering personal and environmental risk;
  • a validated assessment tool, which considers a broad range of potential fall hazards;
  • a formal, observational evaluation of the functional capacity of the person within the context of their environment; and
  • adequate follow‐up planned and support identified for adaptations and modifications.
  In addition, it was deemed good practice to actively involve the older person in the assessment and priority‐setting process.
  The chart audit was used to gauge current practice with high‐ri fall risk clients, the degree of fidelity of intervention delivery to training received, penetration, and the extent to which the practice change was sustained (Gillespie et al., 2012). Charts were included for clients who were 65 years of age and over, were potentially at high risk of falls, received an occupational therapy service, and attended the service within the audit time frame. The baseline audit timeframe was 1 January 2019 to 30 June 2019, the during‐implementation timeframe was 1 June 2020 to 31 December 2020, and the post‐implementation timeframe was 1 January 2021 to 31 December 2022. Audit data were entered into an Excel spreadsheet with notes regarding interpretation and to highlight additional observations made during the audit process.
3. A cross‐sectional staff survey—before and after implementation—was based on the principles of the i‐PARIHS framework (Harvey & Kitson, 2016). The survey comprised 12 questions, with sub‐questions, examining occupational therapists' knowledge, attitudes, confidence, and experience of EAM for fall prevention. The survey was piloted via stage 1 of this research programme (Figure 1) (Pighills, Furness, et al., 2019). An external panel of occupational therapists checked content and readability (Green & Thorogood, 2018). The survey was hosted on the SurveyMonkey platform. Occupational therapists whose role did, or could, include EAM for fall prevention in older people were invited to participate in the pre‐implementation and post‐implementation surveys.
4. A cross‐sectional staff survey—before and after implementation—was based on the principles of the i‐PARIHS framework (Harvey & Kitson, 2016). The survey comprised 12 questions, with sub‐questions, examining occupational therapists' knowledge, attitudes, confidence, and experience of EAM for fall prevention. The survey was piloted via stage 1 of this research programme (Figure 1) (Pighills, Furness, et al., 2019). An external panel of occupational therapists checked content and readability (Green & Thorogood, 2018). The survey was hosted on the SurveyMonkey platform. Occupational therapists whose role did, or could, include EAM for fall prevention in older people were invited to participate in the pre‐implementation and post‐implementation surveys.

The primary outcome for the quantitative study was penetration (integration of the intervention into existing practice), measured over a 30‐month period via chart audit. Secondary outcomes included fidelity, acceptability, and sustainability (Table 1) (Proctor et al., 2011).

**TABLE 1:** Data collection matrix (Proctor et al., 2011).

Outcomes	Indicators	Data source/instrument	When?
1. Penetration (integration of an intervention into existing practice)	Percentage of clients at high risk of falls, referred to community health, who received a WeHSA	Chart audit	During and after implementation
2. Fidelity (extent to which the intervention was implemented as planned)	Proportion of clients (at high risk of falls who received EAM) whose medical records indicated that the intervention delivered was high intensity and falls specific	Chart audit	During and after implementation
3. Acceptability (perception among stakeholders that the intervention is satisfactory)	Staff perceptions, attitude, and confidence about providing EAM	Staff surveys Qualitative study (reported elsewhere)	Baseline and after implementation
4. Sustainability (maintenance of implementation)	Percentage of clients at high risk of falls, referred to community health, who receive a WeHSA after implementation (1/1/2021–31/12/2022)	Chart audit	After implementation

Abbreviations: EAM, environmental assessment and modification; WeHSA, Westmead Home Safety Assessment.

### 2.6 Data analysis

Descriptive statistics were used to summarise the survey and chart audit data reflecting percentages, central tendency, and data spread. Non‐parametric inferential statistics were used to make pre‐implementation and post‐implementation comparisons using IBM SPSS v.22 software (IBM, Armonk, NY, USA). The Mann–Whitney *U* test compared independent distributions of the pre‐implementation and post‐implementation questionnaire variables, as the dependent variables were non‐normally distributed scale data. Spearman's rank correlation coefficient measured the strength and direction of association between non‐normally distributed scale data when comparing the Falls Risk for Older People in the Community screen (FROP‐COM screen) (Russell et al., 2009) and study risk screen results. Chi‐squared and Cramér's *V* tests determined the significance and strength of the association between falls and subsequent death by June 2024 and falls and the number of fall risk factors.

### 2.7 Ethics

The study was granted ethics approval LNR/2019/QTHS/51590 from the Townsville Hospital and Health Service Human Ethics Committee.

## 3 RESULTS

### 3.1 Implementation process and evaluation

Nine occupational therapists participated in the project. Baseline, structured observations of occupational therapy fall prevention practice were carried out (n = 7) in September 2019 to gauge current practice and inform training. The occupational therapists observed demonstrated a mean of 37% adherence to best practice on a scale of 0–3 for 12 items (0 = assessment item omitted; 1 = some elements of the assessment item observed; 2 = most elements of the assessment item observed; and 3 = all elements of the assessment item observed). All seven observations attained a median score of one.

The items that achieved the highest scores were ‘fall history obtained’ and ‘received adequate follow‐up’. The most common low‐scoring items were ‘pre‐assessment tests completed (functional vision, cognition, mobility, and balance)’, ‘use of a validated assessment’, and ‘evidence of client engagement in hazard identification, solution generation, and mutually agreeing recommendations’.

A baseline questionnaire and post‐implementation follow‐up questionnaire were completed by an unpaired sample of occupational therapists, eliciting 16 responses for each timeframe. Questionnaires were sent to all occupational therapists in the health service, whose role could include fall prevention in older people (n = 40), regardless of whether they received training or participated in the implementation project, to establish whether cascade training had taken place. All respondents were women, with baseline and follow‐up means of 10.73 (SD 9) and 7.6 (SD 8.4) years of post‐qualification experience respectively and 31% worked in rural locations within the regional hospital and health service.

There were no statistically significant differences in scores between the two timepoints for knowledge (Mann–Whitney *U* 167, P = 0.149), attitude (Mann–Whitney *U* 138, P = 0.724), confidence (Mann–Whitney *U* 135, P = 0.809), or experience (Mann–Whitney *U* 163, P = 0.196). Median scores increased slightly between baseline and follow‐up (Table 2). However, these domains showed ceiling effects because staff had high levels of knowledge of EAM and fall prevention, had a positive attitude towards the intervention, felt confident in its delivery, and had experience in fall prevention at baseline, leaving little room for improvement and suggesting that environmental assessment for fall prevention was an intervention they were comfortable and familiar with.

**TABLE 2:** Staff survey scores.

Domains	Baseline median score (range)	Follow‐up median score (range)	Test statistic	P value
Knowledge of EAM (maximum score 28)	22.5 (10)	24.5 (12)	*U* 167	0.149
Attitude towards implementation (maximum score 7)	6 (2)	6.5 (4)	*U* 138	0.724
Confidence in delivering EAM (maximum score 5)	5 (2)	5 (5)	*U* 135	0.809
Experience of working in fall prevention (maximum score 6)	4 (5)	4.5 (6)	*U* 163	0.196

Abbreviation: EAM, environmental assessment and modification.

A 1‐day EAM workshop was delivered following baseline observations and staff questionnaires. The workshop was delivered face‐to‐face and via video conference in November 2019 to all community occupational therapists (n = 8). Following training, occupational therapists were given free access to online training modules to refresh and reinforce learning (https://fallspreventiononlineworkshops.com.au/).

Implementation start was planned for early 2020 but was delayed until 1 June 2020 due to the COVID‐19 pandemic. After training, the first implementation step was to establish a referral review process to identify older people likely to be at high risk of falls referred to the service. This was achieved by senior clinicians in the team scheduling time to review clinical information in referrals and categorising clients as either routine or potentially high fall risk, according to the criteria identified by Lusardi et al. (2017) as discussed above. The time allotted by administrative staff for an occupational therapy environmental assessment for potentially high‐risk older people was increased from 60 to 90 minutes to accommodate additional time taken for the assessment. In addition, to identify clients at high risk of falls and to serve as a continuous prompt for clinicians, risk screening tick boxes were added to the occupational therapy home safety assessment form, the client progress notes in the integrated electronic medical record (iEMR), and the multidisciplinary team (MDT) care plans. Inclusion in the care plans stimulated MDT discussion and often triggered new fall prevention referrals. Upon implementation, the occupational therapists changed their initial assessment process whereby the first questions they asked aimed to determine fall risk through screening, using the study fall risk criteria. This practice change was supported by amending clinical forms and iEMR templates.

During the early stages of implementation, the fall risk screen that was already being used within the service—the FROP‐COM screen—and the implementation study fall risk criteria were both recorded in clients' charts. Analysis revealed a statistically significant association between the two screens when the person possessed ≥4 of the five implementation risk factors (Spearman's *ρ*, one‐tailed 0.49, P < 0.001) or ≥3 implementation risk factors (Spearman's *ρ*, one‐tailed 0.64, P < 0.001). This provided informal validity for the implementation project fall risk screen, demonstrating that it identified older people at highest risk.

During and after implementation, structured observations were carried out by peers via the supervision process through work shadowing, with post‐observation reflections discussed during the journey back to the office and formal feedback provided via supervision sessions.

**FIGURE 3:**
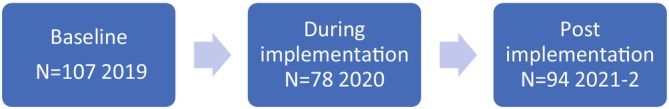
Chart audit details.

A chart audit was completed at baseline for 6 months (from 1 January 2019 to 30 June 2019) (n = 107). A 6‐month during‐implementation chart audit (from 1 June 2020 to 30 November 2020—n = 78) and a 24‐month post‐implementation audit (from 1 January 2021 to 31 December 2022—n = 94) were also carried out (Figure 3).

Results revealed a higher‐than‐average rate of falls in the chart audit cohort; 71% of the 279 cases audited sustained ≥1 fall in the preceding year, and 52% sustained ≥2 falls. Of note, 42% of older people who experienced ≥2 falls in the preceding year (recurrent falls) and 60% of people with ≥3 of the five risk factors died during the 3‐year study period (2019–2022). Indeed, there was a moderate, non‐significant correlation between death and the number of reported falls in the previous year (*χ* ^2^, 21.60 [two‐tailed], P = 0.06, Cramér's *V* 0.287) and a moderate significant correlation between death and the number of fall risk factors identified via the screening process (*χ* ^2^, 151.74 [two‐tailed], P < 0.001, Cramér's *V* 0.381).

Of the 279 older people whose charts were audited, 28% (n = 75) possessed ≥4 risk factors. Of these, 100% were over 65 years old and had a history of ≥2 falls, 77% had mobility and/or balance deficits, 47% were dependent in personal ADL, and 29% expressed fear of falling.

### 3.2 Implementation outcomes

#### 3.2.1 Penetration

As a measure of penetration, when the stringent fall risk criteria adopted for this study were applied, a WeHSA was delivered when indicated in 80% of cases in the during‐ and post‐implementation audits, and 89% of clients did not receive a WeHSA when it was not indicated. When the medical chart auditor applied clinical judgement rather than stringent risk criteria, these figures changed to 55% of cases receiving a WeHSA when indicated and 96% not receiving a WeHSA when not indicated. This disparity is due to clients who sustained serious fall‐related injuries or fractures but only had a history of one fall in the preceding year or clients being referred for occupational therapy fall prevention intervention and not being offered the WeHSA because they did not meet the study fall risk criteria.

#### 3.2.2 Fidelity

The extent to which the intervention was implemented as planned was assessed via the chart audit, which revealed that in 100% of cases, across all occupational therapists, when EAM was delivered, a high‐intensity intervention was provided. All four criteria, used to judge intervention intensity, were consistently met. However, the optional fifth criterion of actively involving the older person in the assessment and priority‐setting process was rarely documented; therefore, it was impossible to discern whether this criterion was achieved.

#### 3.2.3 Acceptability of the intervention

Staff attitude towards the intervention and confidence in delivering it were measured via staff surveys and further explored by examining staff perceptions and reflections on the implementation process as a component of the phase 2 qualitative element of this study (reported elsewhere). As reported above, staff knowledge, attitude, and confidence were high or positive from the outset, leaving little room for improvement and creating a result ceiling effect. Therefore, there were no statistically significant changes in knowledge, attitude, and confidence scores.

#### 3.2.4 Sustainability

To sustain the practice change, EAM was included as a standing agenda item in staff meetings, and online training was funded for all new occupational therapists as a component of their orientation process. New staff were encouraged, and allocated protected time, to complete the online training. They observed existing occupational therapists carry out 1–2 WeHSAs and were then observed leading the assessment on 1–2 occasions. As stated above, the inclusion of fall risk screening prompt questions/tick boxes on the clinical assessment forms, iEMR documentation templates, and MDT care plans served as consistent reminders for clinicians.

The chart audit also provided evidence that the fall prevention training was being generalised to clients who did not meet the criteria to receive a WeHSA. General fall prevention advice and specific advice on areas such as footwear, environmental design, vision, lighting, and colour contrast were evident in occupational therapy entries in clients' charts.

Results showed that occupational therapists who did not attend the baseline EAM training delivered the WeHSA to 22% of the clients who received it. This demonstrates that cascade training was embedded in the service. In addition, the audit revealed that 2 years after implementation, 34% of older people referred to occupational therapy who were potentially eligible received a WeHSA and that 97% of older people received the intervention when indicated, with 95% not receiving the intervention when not indicated. This sensitivity measure increased from 80% during implementation, suggesting that occupational therapists became more adept at targeting the intervention towards those in need. Indeed, at the end of 2024, implementation was still being sustained, with expectations being set during orientation and monitored via supervision sessions.

## 4 DISCUSSION

This implementation study presents the results of embedding best practice EAM in a regional health service in Queensland from June 2020 to December 2022. Penetration was the primary measure of implementation, and results revealed an 80% penetration rate during implementation, increasing to 97% in the post‐implementation chart audit. We had anticipated that implementation would tail off as time passed; however, diffusion of innovation theory suggests that, if innovation to practice is salient to the context and audience, uptake will increase and become more embedded over time (Rogers, 2003). Indeed, staff report that to date, 55 months after implementation, the intervention is sustained. This is likely because steps were taken to align facilitation of the practice change to the needs of the team members within their specific context, which supported success in implementation (Harvey & Kitson, 2016). Contextually driven systems and processes were established, with all new starters being inducted, trained, and mentored to implement the intervention. In addition, implementation took place in a single, small, cohesive team of early career occupational therapists who were proactive in introducing service improvements, supported one another and provided mentorship for new starters. Facilitation within the organisation was central to the process, with implementation supported by effective and engaged leadership both within the team and occupational therapy service as a whole (Harvey & Kitson, 2016). Furthermore, experienced team members acted as strong opinion leaders supporting the diffusion of EAM throughout the team, reaching those who may have been reluctant to adopt the change (Rogers, 2003). In contrast, implementation was unsuccessful at a second site (reported elsewhere), which did not progress with implementation beyond the baseline practice audit and initial training (Tynan et al., 2022). Implementation in the unsuccessful site focussed on one individual providing the intervention without peer, structured team, or leadership support. In addition, as this site was closer to a metropolitan area, restrictions on home visiting, imposed due to the COVID‐19 pandemic, were too prohibitive to enable the site to progress implementation.

Baseline structured observations concurred with the findings of the chart audit conducted in stage 1 (reported elsewhere), which was that the intervention was not implemented in practice prior to the implementation project (Pighills, Furness, et al., 2019). Areas of good practice for the successful site at baseline included fall history taking, conducting a standardised functional assessment of the client in their home environment, and providing written recommendations and adequate follow‐up. However, there was no evidence of the occupational therapists using any validated fall prevention assessments; administering pre‐assessment tests of functional vision, functional cognition, mobility, and balance; or engaging the client in identifying fall hazards, developing/owning solutions, and mutually agreeing on recommendations. In addition, there was rarely documented evidence that fear of falling had been assessed. There exist validated tools that are quick and simple to administer, including the yes/no response question ‘Are you afraid of falling?’ and the Falls Efficacy Scale—International (FES‐I) version, short form, which comprises four simple questions (Denkinger et al., 2008; Kempen et al., 2007). Either of these screens could be added as a prompt to occupational therapy home assessment templates and electronic medical record forms and would take minimal time to administer. Along with the work already completed in identifying the intervention, place, and people involved, the baseline audit proved useful for understanding the pre‐implementation processes, thus providing focus for targeted implementation strategies that aimed to support adoption of these missing elements. The type of insights drawn from this preliminary work has been shown to be central to SI (Wilson & Kislov, 2022).

The staff questionnaire revealed high levels of knowledge of EAM, a positive attitude, high confidence levels, and experience in the care of older people and fall prevention at both timepoints, slightly increasing at follow‐up. However, a lack of sensitivity of the questionnaire and the evident ceiling effect prevented meaningful analysis in these domains. It is not surprising that these four domains scored highly at baseline, as EAM is a core occupational therapy skill. The baseline positivity towards the change and familiarity with EAM may have acted to decrease the perceived risk, therefore facilitating implementation (Wejnert, 2002).

Our results revealed a higher fall rate than is often reported, consistent with other studies (Cai et al., 2023; Pighills et al., 2011). Indeed, the fall rate in this potentially high‐risk cohort of older people was double that reported in the literature, with 71% experiencing ≥1 fall in the preceding year and 52% reporting ≥2 falls (Campbell & Robertson, 2007; Gillespie et al., 2012). These fall rates were in the cohort of older people who underwent referral review and were offered longer initial appointment times before the occupational therapist had screened their fall risk against the stringent study criteria. This elevated fall rate might have partially contributed to the 60% death rate (during the 3‐year study period) in older people with ≥3 of the five risk factors, due to the association between falls and subsequent death within a year (Scuffham et al., 2003; Stevens et al., 2014).

Of the older people who possessed ≥4 fall risk factors, aside from them all being aged ≥65 with a history of ≥2 falls, mobility and/or balance deficits were the most frequent risk factor (77%), followed by dependence in personal ADL (47%), and lastly fear of falling (29%). However, this figure is unlikely to reflect the prevalence of fear of falling, as a measure of fear of falling was rarely documented in clients' charts. Mobility and balance deficits often precipitate, and in many cases cause, dependence in personal ADL; therefore, we would have expected this risk factor to be the most prevalent, as was the case.

### 4.1 Occupational therapy screening process

The FROP‐COM screen was being used prior to implementation to identify fall risk. This screen was discontinued once the risk screening process for the project was established. However, there was a crossover period that provided a useful mechanism to validate risk criteria used for the project by enabling a comparison of scores from both screening processes. This comparison provided validation of the study risk screening process demonstrating a statistically significant association between falls and risk factors for the two screens.

A crucial element of implementation was establishing a robust referral review and subsequent fall risk screening process at the outset. The referral review process was based on the information contained in the referral and used to determine whether a 90‐minute appointment was indicated to allow time to complete the WeHSA. However, the referral information was inconsistent; therefore, occasionally 60 minutes were allocated for the initial assessment, but screening suggested high fall risk, indicating that the WeHSA should be used. We could have enhanced the accuracy of the referral review process by educating referrers prior to study commencement and seeking more information for inadequate referrals after implementation, which would coincidentally have reinforced the referrer education process.

To ensure that the highest risk population was targeted, the study team determined that clients should be deemed high risk, thus eligible for EAM, if they possessed four out of five predetermined fall risk factors (see Section 2) (Lusardi et al., 2017; Pighills, Drummond, et al., 2019). It transpired that this criterion was too stringent. Several clients were not offered the intervention because they only met three of the criteria, despite being referred due to an injurious fall (often with an environmental cause), multiple or recurrent falls, or specifically for occupational therapy fall prevention intervention—these factors should automatically trigger EAM regardless of the number of other risk factors present. The occupational therapists used the fall risk screening score to determine the need for EAM in preference to employing clinical reasoning. Indeed, occupational therapists routinely documented the following standard statement in clients' medical records: ‘Consumer meets three out of five risk factors, therefore not meeting criteria for high falls risk. Westmead Home Safety assessment was therefore not completed.’ However, the chart audit revealed that, although the assessment was not completed when indicated in some cases, components of EAM were delivered to clients who were not categorised as high fall risk based on the education and training provided to the clinicians.

Listing the five risk factors as a tick box fall risk screen at the top of the occupational therapy home safety assessment before implementation was a vital step to ensure that fall risk screening was carried out. The use of this type of computerised reminder as an implementation strategy has been demonstrated to elicit a small to moderate improvement in clinical behaviours (Shojania et al., 2009). As an afterthought, post‐implementation, the same tick box risk screen was added to the iEMR. This was a useful measure to ensure auditor accuracy in identifying high‐risk older people in the chart audit. In addition, fear of falling was rarely assessed or even mentioned in clients' occupational therapy or medical records. Therefore, for the purposes of the chart audit, in most cases, it was not possible to determine whether this risk factor was present until the tick box risk screen was added to iEMR.

### 4.2 Learnings

Future implementation of the EAM process should start at the beginning of the referral continuum and include educating referrers on critical information required and requesting more information or rejecting inadequate referrals to reinforce referrer education.

Occupational therapists need prompting to assess older people for fall risk. Future studies should consider including a tick box risk screen in the client's initial assessment, supporting occupational therapists to use clinical reasoning to determine the need for EAM, and encouraging them to err on the side of caution by potentially using the short form WeHSA for clients with fewer risk factors whom they deem would benefit from the intervention (Clemson, 2018). In addition, relaxing the risk factor eligibility to ≥3/5 implementation risk factors, with age ≥65 and a history of ≥2 falls and/or an injurious fall in the past year and/or referred to occupational therapy for fall prevention as required criteria plus one additional risk factor, would capture more older people who would benefit from the assessment while still ensuring that intervention recipients are at high fall risk.

The WeHSA was completed on the day of the initial assessment, immediately after fall risk screening. This ensured that the older person received the WeHSA and was potentially a good strategy to ‘strike while the iron is hot’. Occupational therapists scheduled more 90‐minute appointments immediately after implementation than they did in subsequent years. Incrementally, the number of scheduled 90‐minute appointments declined, which suggests that there is a ‘window of opportunity’ to provide additional training and support immediately after implementation to keep up the momentum. Indeed, the i‐PARIHS framework espouses the need for ongoing facilitation during and after implementation to embed the service change (Harvey & Kitson, 2016). In this study, the occupational therapists voiced the need for refresher training, as the clinicians that were originally part of the project had significant ‘buy‐in’, thus, a vested interest in keeping the intervention going; however, with staff turnover, motivation might dwindle and implementation tail off if ongoing training is not provided.

The occupational therapists became more discerning in reviewing referrals and determining the likelihood of the client meeting the stringent risk criteria as the study progressed. In fact, the referral review and screening process became more refined with time, with results revealing that the number of people who should have received a WeHSA but did not get one was only 3% after implementation. This suggests that occupational therapists only provided the WeHSA to those older people at high risk of falls, as per protocol.

It would have been useful for the chart audit for the WeHSA to be uploaded to the iEMR or at least to identify in iEMR that the WeHSA had been completed, which is what the occupational therapists did in their progress notes. This would alert the auditor to the existence of the WeHSA and keep all records in one place, including the WeHSA outcome and results. The chart audit revealed that occupational therapists did not re‐assess risk as the client's condition changed over time. Mechanisms need to be put in place to routinely review fall risk scores when clients are re‐assessed. In addition, the intervention was implemented in one service, but the intervention was not followed through to other services even when the same occupational therapist was working across both services, suggesting that occupational therapists might need more support to generalise the intervention to other settings.

### 4.3 Limitations

As is the case with implementation research in general, the research design was subject to bias; however, the research goal was to evaluate the implementation process, not to examine the effectiveness of the intervention (Peters et al., 2013). Measures were introduced to ensure rigour, such as using an objective measure for the primary outcome, completing the medical chart audit via a researcher rather than a clinician, using a structured audit tool, and adopting specific criteria to determine the intensity and quality of the intervention delivered.

The staff questionnaire was sent to all occupational therapists in the service who worked with older people and thus may be involved in fall prevention. However, this produced unpaired pre‐questionnaire and post‐questionnaire data, which would have diluted the implementation effect, thus making it more difficult to detect a statistically significant difference between timepoints. The rationale for widening the sampling frame to include occupational therapists outside of the team in which implementation was taking place was to provide statistical power and to detect whether the training and implementation were disseminated more widely than the immediate team. The questionnaire needs refining for future use to increase sensitivity or removal from the process, as it did not add value to the study.

Home visit restrictions, imposed due to COVID‐19, resulted in a 6‐month delay to the start of the project. Ongoing restrictions prevented investigators from carrying out structured observations of occupational therapy practice during implementation. Therefore, these observations were carried out by peers and/or the nature and content of the assessment were addressed through the established supervision process.

Significant team waitlist stressors and time pressures limited capacity to provide follow‐up home visits, if indicated. To mitigate this, follow‐up visits were delegated to the occupational therapy assistant, as appropriate, in addition to all the 8‐ to 12‐week follow‐up telephone calls.

GEAT2GO (Goods, Equipment and Assistive Technology) was introduced within the team in August 2021. This programme aims to maintain/increase Commonwealth Home Support Program clients' independence through funded equipment and technology provision (Commonwealth of Australia, 2025). Most items require an occupational therapy prescription; thus, the team's clinical burden increased dramatically after the GEAT2GO rollout. The project impact of this additional workload pressure has been increasing instances where the WeHSA cannot be completed due to time constraints. However, access to these items, free of charge to clients, assisted in remediating some fall hazards identified during the WeHSA.

## 5 CONCLUSION

This implementation research study demonstrated that occupational therapy‐led EAM was successfully implemented, delivered as per protocol, embedded in practice, and sustained beyond the end of the 30‐month follow‐up period.

SI required occupational therapy staff motivation to adopt the best practice intervention, support systems in place (e.g., organisational and management support, referral review, extended appointment scheduling, fall risk screening, and supervision), new documentation processes, a network of peer support, and a cascade training process established. After implementation, clinicians became more discerning in targeting the intervention towards those at high risk of falls, and there was evidence of new starter occupational therapists delivering the intervention, indicating that cascade training was established. Results also showed that the fall prevention intervention was generalised and influenced practice with older people who were not deemed high fall risk.

The study provided invaluable learnings about the research design and implementation process that should be incorporated in a national implementation project, which commenced in 2025.

## AUTHOR CONTRIBUTIONS

AP and AT developed the research concept and coordinated the data collection with assistance from TB and AE. AP completed the data entry, analysis, and initial drafts of the manuscript. All authors contributed to the discussion, provided critical review of the manuscript drafts, and have read and approved the final version of the manuscript.

## CONFLICT OF INTEREST STATEMENT

The authors declare no conflicts of interest.

## Data Availability

The data that support the findings of this study are available on request from the corresponding author.
